# Bioinspired Computation for Identifying Joint Compliance in Biomimetic Flexible Manipulators

**DOI:** 10.3390/biomimetics11070474

**Published:** 2026-07-07

**Authors:** Abdelraheim Emad Abdelraheim, Mohamed Nejlaoui, Nasser Ayidh Alqahtani

**Affiliations:** Department of Mechanical Engineering, College of Engineering, Qassim University, Buraida 51452, Saudi Arabia; aahmad@qu.edu.sa (A.E.A.); m.nejlaoui@qu.edu.sa (M.N.)

**Keywords:** Genetic algorithms, manipulators, stiffness, finite element analysis, optimization

## Abstract

High-precision robotics is frequently compromised by joint compliance, a factor often over-simplified by traditional rigid-body modeling. This research investigates the structural dynamics of a two-link manipulator, addressing critical discrepancies between experimental data and conventional models. Much like biological musculoskeletal systems, joint flexibility fundamentally influences the dynamic response of articulated structures. While traditional rigid-joint models accurately capture mode shapes, they yield excessive natural frequency prediction errors with peaks reaching 72%. To bridge this gap, a refined Flexible-Joint Finite Element Model (FJFEM) is developed to mimic adaptive joint compliance. This model is integrated with a bio-inspired computational framework (a Double-Stage Genetic Algorithm Framework (DSGAF)) to identify configuration-dependent joint stiffness across the operational workspace, where experimental frequencies $f_{1}$ and $f_{2}$ shift nonlinearly from 25.5 Hz to 44 Hz and 92.2 Hz to 51 Hz, respectively. Experimental validation demonstrates that this evolutionary strategy reduces frequency tracking errors to less than 3.5% across all positions, achieving an average identification routine runtime of 1.8 s. By capturing nonlinear compliance behavior, this framework provides a robust foundation for the design, online calibration, and vibration control of advanced flexible robotic systems.

## 1. Introduction

Nowadays, robotic manipulators are more often designed as light and fast systems, following the natural elasticity of the biological musculoskeletal structures [1,2,3,4,5,6]. Early robotics literature focused on rigid-body dynamics to simplify control. But the structural compliance of joints and links is now a critical focus. Flexibility has a significant impact on dynamic performance and accuracy, requiring integrated modeling methods that incorporate the adaptive stiffness exhibited in biological limbs [7,8,9,10].

Foundational research laid the mathematical foundation for the modeling of oscillatory behavior and vibration theory in mechanical systems [11,12,13,14]. Later studies concentrated on localising the influence of drive-train elasticity and link-beam structures on tracking accuracy [15,16,17,18,19,20,21]. To address the complexity of such systems, structural simplifications (e.g., one-link or two-link models) have been widely used in parametric studies to isolate variables such as beam length and mass ratios [22,23,24,25,26,27,28,29,30]. However, conventional predictive methods such as Lumped Mass Models (LMMs) and rigid-joint Finite Element Methods (FEMs) are not always consistent with experimental data [31,32,33,34].

The initial experimental investigations (in this study) showed that rigid-joint models (RJFEMs) can capture mode shapes but generate high natural frequency errors, with peaks of 19.7% and 72%. This divergence confirms a major shortcoming of traditional modeling: the lack of localized, configuration-dependent joint flexibility and its nonlinear interaction with the global structure.

This research proposes a Flexible-Joint Finite Element Model (FJFEM) with a torsional spring to model joint stiffness, thereby mimicking the variable compliance of natural joints and filling this gap. The main novelty of the present work is the creation of a Double-Stage Genetic Algorithm (DSGA) scheme. Unlike previous works that exploit static or trial-and-error stiffness values, the DSGA treats compliance as a dynamic function.

In the first stage, multi-objective Pareto optimization is used to minimize frequency errors at discrete points, and the second stage derives continuous fifth-order polynomial equations to map stiffness along the entire workspace. The implementation of these optimized parameters in this work results in a significant decrease in frequency error (from 72% to 3%) and provides an effective technique for vibration regulation of biomimetic flexible structures.

The proposed compliance model draws inspiration from the way human joints (such as the elbow) rely on muscular co-activation. In nature, antagonistic muscles dynamically regulate joint compliance to absorb shocks and meet variable structural demands. By replacing the traditional rigid-joint assumption with a localized torsional spring model (FJFEM), this work captures how biological limbs leverage soft tissues to maintain structural stability without losing flexibility during multi-axis shifts. In biological musculoskeletal structures, variable stiffness is primarily regulated via the co-contraction of antagonistic muscle pairs, dynamically modulating localized joint compliance to absorb dynamic shock loads and maintain stability across varying geometric topologies. In this work, the parameterization of K(θ) acts as a functional abstraction of this biological co-activation mechanism. By compressing the macroscopic behavior of flexible soft tissues into an adaptive, configuration-dependent localized spring framework, the FJFEM reproduces the structural damping and variable compliance inherent to natural limbs without incurring the intensive computational burden of continuum mechanics. The main contributions of this research can be summarized as follows:

- Flexible-Joint Finite Element Model (FJFEM) Development: Moving from conventional rigid-joint assumptions to a bio-inspired flexible paradigm, incorporating nonlinear localized joint compliance.
- Development of Double-Stage Genetic Algorithm (DSGA) Framework: A new two-level optimization framework. Stage I performs discrete multi-objective Pareto optimization to minimize frequency errors, while Stage II obtains a global, continuous stiffness-to-configuration mapping.
- Comparative Benchmark: The proposed DSGA offers an analytical equation K(θ) under workspace for dynamic control, while the traditional methods (e.g., GA or PSO) acquire static parameters.
- Superior Error Reduction: Demonstrates a reduction in natural frequency prediction error from more than 72% in rigid models to less than 3.5% for all configurations.

To delineate the contributions of this work from the existing literature, Table 1 compares the proposed framework against contemporary state-of-the-art methodologies. While several advanced techniques effectively identify structural and joint parameters, they typically treat joint stiffness as a static value or incur significant computational overhead when evaluating continuous workspace variations. The proposed DSGAF addresses these limitations by mapping configuration-dependent compliance onto continuous polynomial functions, achieving a substantial reduction in predictive frequency errors without expanding computational complexity.

The rest of this paper is organized as follows. Section 2 presents the experimental investigation and hardware setup for modal analysis. Section 3 provides the mathematical analysis and modeling. Section 4 describes the bio-inspired optimization strategy and simulation analysis. Section 5 discusses the results and comparative analysis. Finally, Section 6 concludes the work.

## 2. Experimental Investigation

**Figure 1:**
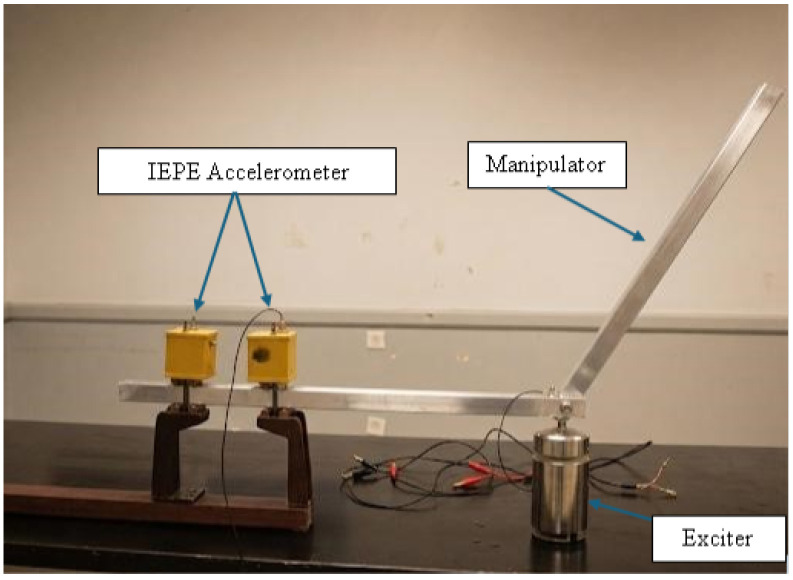
The real experimental manipulator.

**Figure 2:**
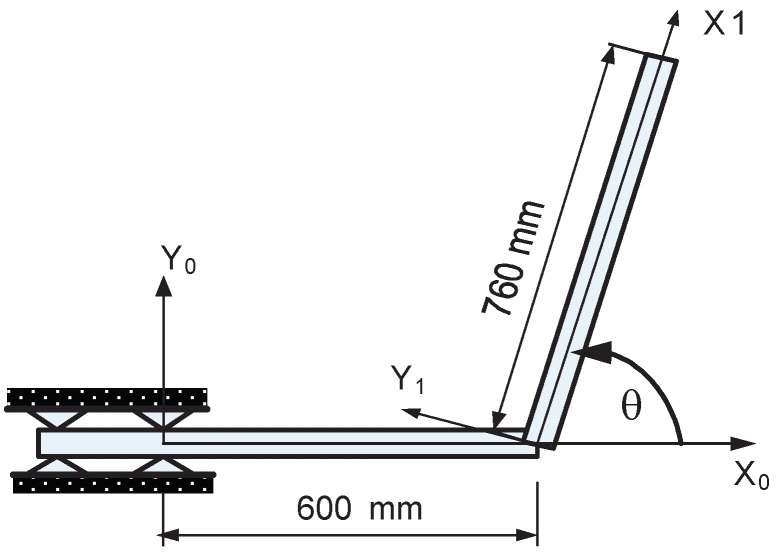
The manipulator model.

Figure 1 and Figure 2 show the experimental two-link manipulator. Fixed securely at its base like a cantilever, the arm has a 60 cm primary link and a 76 cm secondary link. Both links are constructed from hollow square tubing, measuring 40 mm on the outside with a 1.1 mm wall thickness to optimize the weight-to-strength ratio.

The manipulator is built from aluminum (75 GPa Young’s modulus, 2700 kg/m^3^ density). The joint angle (θ) was changed from $0^{\circ }$ to $150^{\circ }$ and its dynamic response was monitored in each angle case.

**Figure 3:**
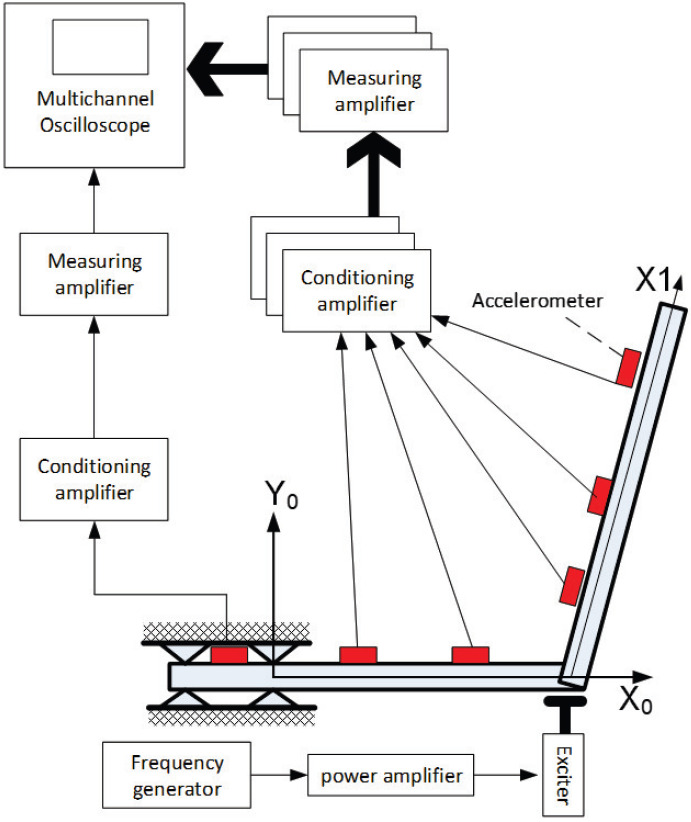
Schematic drawing of the experimental setup.

As presented in Figure 3, the experimental system consists of two main parts: the first part is for excitation, and the second part is for measuring the resulting frequency. The first part consists of a frequency generator, a power amplifier, and an excitation device. The second part consists of accelerometers, conditioning and measuring amplifiers, and an oscilloscope. The characteristics of the experimental components used are summarized in Table 2.

As illustrated in the detailed schematic configuration in Figure 3, the electromagnetic exciter is attached near the end of the first link adjacent to the joint axis to apply a vertical dynamic excitation force to the structure. To capture the resulting dynamic responses, two lightweight IEPE piezoelectric accelerometers are mounted along the links. These sensors are oriented perpendicular to the longitudinal axis of their respective links to isolate and measure lateral bending vibrations. Using two accelerometers simultaneously prevents data loss due to modal node positioning (where a sensor placed exactly at a vibration node would fail to record a specific frequency) and guarantees that both the first and second natural frequencies are fully captured across the entire workspace configuration range. The experimental procedure followed these steps:

1. **Set Configuration:** Fix the manipulator at a specific joint angle θ.
2. **Frequency Sweep:** Excite the system across a broad frequency range.
3. **Data Acquisition:** Capture vibration amplitudes via accelerometers at designated locations.
4. **Signal Processing:** Route signals through conditioning and measuring amplifiers to the oscilloscope.
5. **Peak Identification:** Locate the excitation frequencies that generate maximum vibration amplitudes.
6. **Modal Analysis:** Derive the natural frequency (*f*) and damping factor (ζ) using the half-power bandwidth method [11,14].
7. **Data Collection:** Measure the first four natural frequencies across six manipulator configurations (summarized in Table 3).

To verify the repeatability of the experimental measurements, the natural frequency was measured through five independent tests for each configuration, after which the Standard Deviation (SD) and the coefficient of variation, defined as (CV% = (SD/Mean experimental frequency) × 100), were calculated. The obtained results are summarized in Table 4. It can be observed that the maximum value of the coefficient of variation is less than 0.85% for all tested configurations. Such low CV values indicate that the experimental uncertainty is negligible and confirm the high repeatability and reliability of the experimental measurements.

## 3. Mathematical Analysis and Modeling

### 3.1. The Analytical Formulation (LMM)

**Figure 4:**
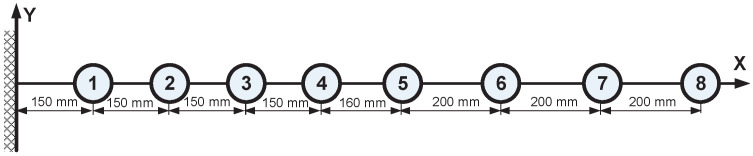
Scheme of the LMM for $\theta =0^{\circ }$.

The LMM is used to assess the experimental results using two configurations: $\theta =0^{\circ }$ and $\theta =90^{\circ }$. The data of the experimental model is used here. As illustrated in Figure 4, each link is discretized into four lumped masses. Lateral vibrations are described by a set of eight generalized coordinates, with one coordinate assigned to each mass. These coordinates are defined perpendicular to the longitudinal axis of their respective links.

#### 3.1.1. Lumped Mass Model (LMM) for $\theta =0^{\circ }$

For the configuration where $\theta =0^{\circ }$, the system’s mass matrix is defined by Equation (1).

(1) $$\left[M\right]=\left[\begin{array}{cccc} m_{1} & 0 & \ldots & 0\\ 0 & m_{2} & \ldots & 0\\ \vdots & \vdots & \ddots & \vdots \\ 0 & 0 & \ldots & m_{8} \end{array}\right].$$

**Figure 5:**
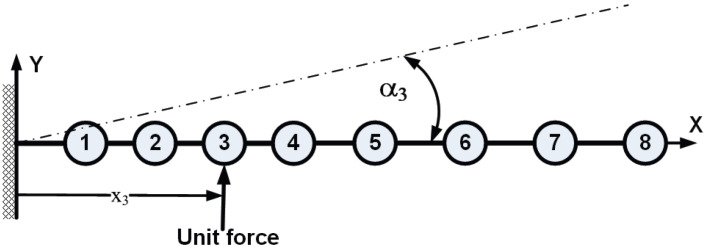
Unit force approach at $\theta =0^{\circ }$.

The symmetric flexibility matrix [*a*] is constructed using the virtual unit force method, which determines the deflection at the *i*th point (defined by $x_{i}$) resulting from a unit force applied at the *j*th point (defined by $x_{j}$). Figure 5 illustrates the idea where angle $\alpha_{3}$ is the slope due to the virtual unit force application, if considered at point $x_{3}$, and is given by [35]:

(2) $$\alpha_{j}=\frac{x_{j}^{2}}{2EI}$$

The individual elements $a_{ij}$ for this setup are derived as follows:

(3) $$a_{ij}=\frac{3x_{j}x_{i}^{2}-x_{i}^{3}}{6EI},\;i\leq j\;\forall \;i,j$$

(4) $$a_{ij}=\frac{3x_{j}^{2}x_{i}-x_{j}^{3}}{6EI},\;i\geq j\;\forall \;i,j$$

#### 3.1.2. Lumped Mass Model (LMM) for $\theta =90^{\circ }$

**Figure 6:**
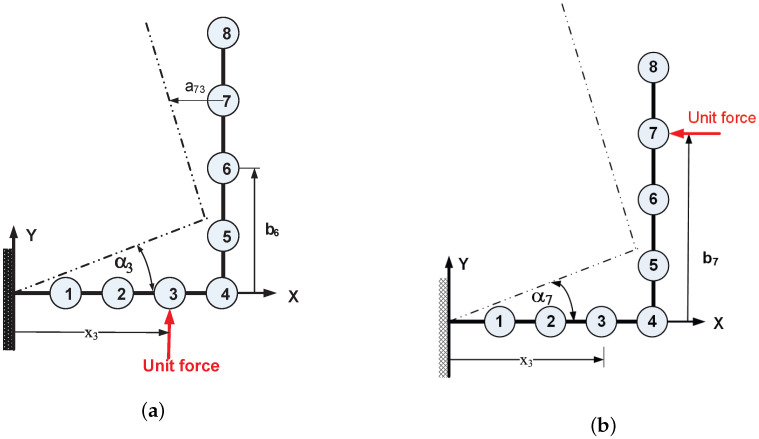
Scheme of the LMM for θ=π/2. (**a**) Unit force is at $m_{3}$. (**b**) Unit force is $m_{7}$.

In this configuration, the second link is perpendicular to the first (Figure 6). While the mass matrix from Equation (1) remains applicable, the flexibility matrix calculation is divided into two distinct scenarios based on where the unit force is applied.

**A. Virtual Unit Force Applied on the First Link ($x_{j}$, where 1≤i≤4)**

When the unit force is on the first link, the slope angle is defined as given in Equation (2).

- **Deflection on the First Link (1≤i≤4):** Elements $a_{ij}$ are calculated using the standard beam Equations (3) and (4).
- **Deflection on the Second Link (i>4):** If $b_{i}$ is defined as shown in Figure 6a, the lateral deflection is given by
  (5) $$a_{ij}=\alpha_{j}\;b_{i}$$

**B. Virtual Unit Force Applied on the Second Link (j>4)**

A unit force on the second link at distance $b_{j}$ from the second joint creates a moment $M_{j}$ and a slope angle $\alpha_{j}$ on the first link:

(6) $$M_{j}=b_{j}$$

(7) $$\alpha_{j}=\frac{M_{j}x_{4}}{EI}=\frac{b_{j}x_{4}}{EI}$$

**Figure 7:**
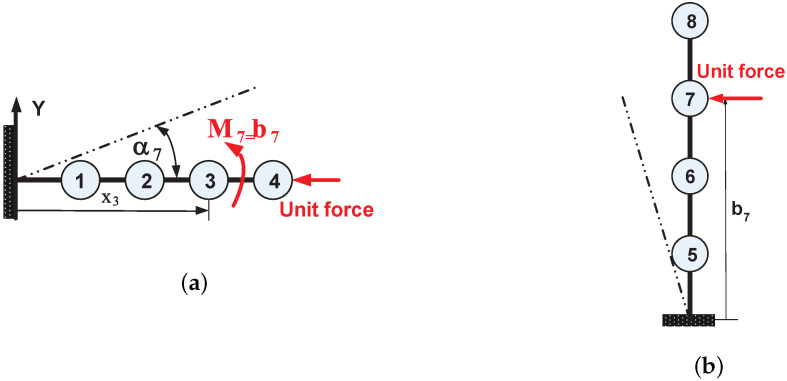
Case of deflection on the second link (j>4). (**a**) Second link as a rigid body. (**b**) Second link as a cantilever beam.

For example, Figure 7 shows the case where the unit force is applied at $x_{7}$, while the deflection angle is situated on the first link.

- **Deflection on the First Link (**1≤i≤4**):** The deflection at $x_{i}$ is given by
  (8) $$a_{ij}=\frac{b_{j}x_{i}^{2}}{2EI},\;i\leq 4$$
- **Deflection on the Second Link (**i>4**):** This case is solved via superposition of two steps.

**Step A:** Treating the second link as a rigid body attached to the end of the first link (Figure 7a). In this part, the unit force is considered as transmitted to the mass at $x_{4}$ with an associated torque of moment $M_{j}$ (as in Equation (6)). This yields a baseline deflection:

(9) $$a_{ij_{\left(1\right)}}=\alpha_{j}b_{i}=\frac{b_{i}x_{4}b_{j}}{EI},\;i>4,\;j>4$$

**Step B:** Treating the first link as fixed and the second link as a cantilever beam (Figure 7b). The corresponding lateral deflection at the second link element can be computed using formulas similar to those shown in Equations (3) and (4).

Combining these steps, the final elements are:

(10) $$a_{ij}=\frac{b_{i}x_{4}b_{j}}{EI}+\frac{3b_{i}^{2}b_{j}-b_{i}^{3}}{6EI},\;i\leq j$$

(11) $$a_{ij}=\frac{b_{i}x_{4}b_{j}}{EI}+\frac{3b_{i}b_{j}^{2}-b_{j}^{3}}{6EI},\;i\geq j$$

To not lose the generality, the elements of the flexibility matrix (for the LMM) when $\theta =90^{\circ }$ are tabulated in Appendix A.

The first calculated four natural frequencies for both manipulator configurations ($\theta =0^{\circ }$ and $\theta =90^{\circ }$) are presented in Table 5. The data clearly indicates that these natural frequencies are configuration-dependent, shifting significantly as the joint angle changes.

The same methodology may be systematically applied to derive the full symmetric flexibility matrix for any other manipulator configuration (different angle θ).

### 3.2. Rigid-Joint Finite Element Modeling (RJFEM)

**Figure 8:**
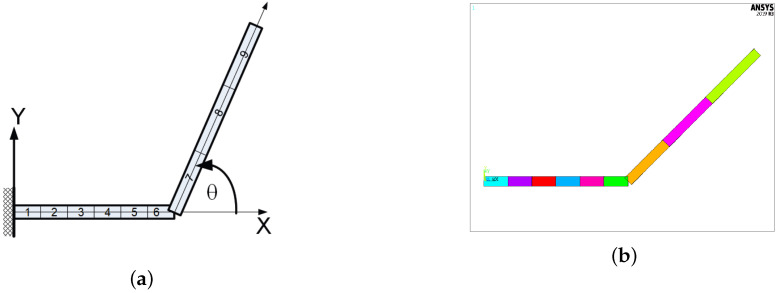
Model RJFEM.

In this phase of the study, the ANSYS 2018 software package [36] was utilized to model the manipulator model. The data of the experimental model is used here. As illustrated in Figure 8, the finite element model consists of nine elements. The BEAM3 element type was selected from the ANSYS element library for this task. BEAM3 is a two-node uniaxial element that provides capabilities of tension, compression, and bending [36]. Each node within this element features three degrees of freedom: translations in the nodal *x* and *y* directions and rotation about the *z* axis.

**Figure 9:**
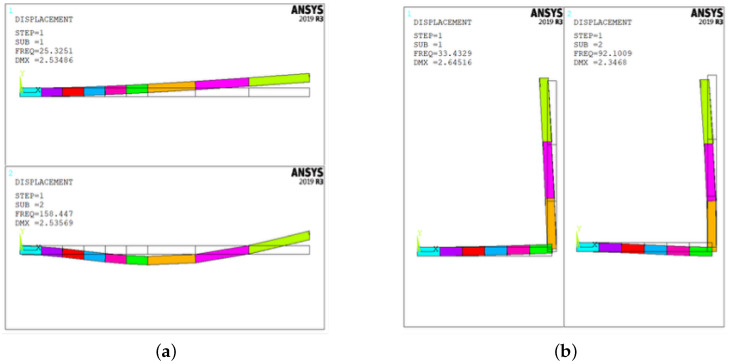
Examples of the mode shapes out of the RJFEM.

The RJFEM assumes a fixed connection at the root and a perfectly rigid connection between the two links. The RJFEM successfully captures the fundamental and higher-order mode shapes for the system at specific configurations. These results provide a baseline for the global system matrices, allowing for a direct comparison between the theoretical rigid behavior and the observed experimental data. Figure 9 illustrates some of the obtained mode shapes for the model under the assumption of perfectly rigid joints. Table 6, however, lists the resulting natural frequencies out of the RJFEM.

The first four natural frequencies for the manipulator model under investigation were calculated according to Blevins [34]. The Blevins’s formula for the *i*th natural frequency of a clamped-free beam given in Table 3 in [34] is:

(12) $$f_{i}=\frac{\lambda^{2}}{2\pi L^{2}}\sqrt{\frac{EI}{\bar{m}}}$$

This formula works only for the full extended ($\theta =0^{\circ }$) manipulator. The Blevins’s tabulated parameter λ is given in numeric values in [34]. The modulus of elasticity is given by *E*, and *I* is the area moment of inertia about the neutral axis. The length of the beam is denoted by *L*. The symbol $\bar{m}$, however, is the mass per unit length of the beam. Substituting by the beam data, the Blevins’s formula is reduced to $f_{i}=7.2078\;\lambda^{2}$. The Blevins’s parameter λ and the corresponding natural frequencies are tabulated in Table 7.

### 3.3. The Need for More Investigation

**Figure 10:**
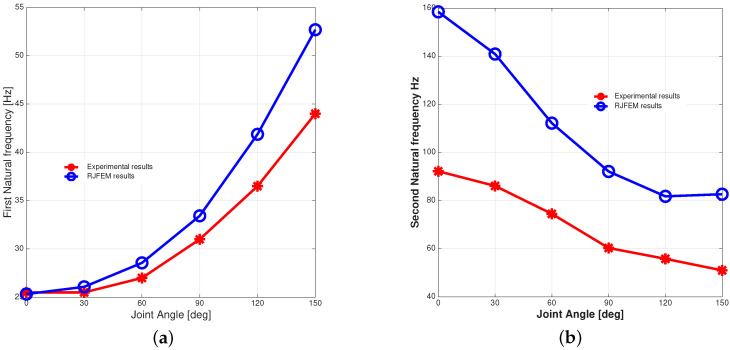
Evolution of the first two natural frequencies as a function of θ. (**a**) First natural frequency. (**b**) Second natural frequency.

A comparison between all results (previously listed in Table 3, Table 4, Table 5, Table 6 and Table 7) may lead to the following summary and conclusions:

(a) *Summary:*

- The results obtained from the experimental work and the RJFEM give the natural frequencies for $\theta =30^{\circ }$, $60^{\circ }$, $90^{\circ }$, $120^{\circ }$ and $150^{\circ }$.
- The LMM gives natural frequencies only for $\theta =0^{\circ }$ and $90^{\circ }$.
- Based on Blevins, only natural frequencies for $\theta =0^{\circ }$ are available.

(b) *Observations:*

- In general, the natural frequencies depend on the manipulator configuration.
- Given the dominance of the first and second natural frequencies in dynamic analysis, this study focuses on the comparison of their behavior, as shown in the following.
- Figure 10 shows the results from the experimental work and the RJFEM only. The figure illustrates the frequencies across all the manipulator model configurations.
- The main observation is the divergence in modal frequency behavior as the manipulator configuration changes, showing that the first natural frequency follows an upward trend while the second natural frequency exhibits a continuous decrease as the joint angle θ increases from $0^{\circ }$ to $150^{\circ }$.
- Based on the validated experimental data, $f_{1}$ begins at 25.5 Hz at the fully extended position ($0^{\circ }$) and rises to 44 Hz at $150^{\circ }$, whereas $f_{2}$ starts at a maximum of 92.2 Hz and drops significantly to 51 Hz over the same range.

(c) *Result Comparison:*

**Figure 11:**
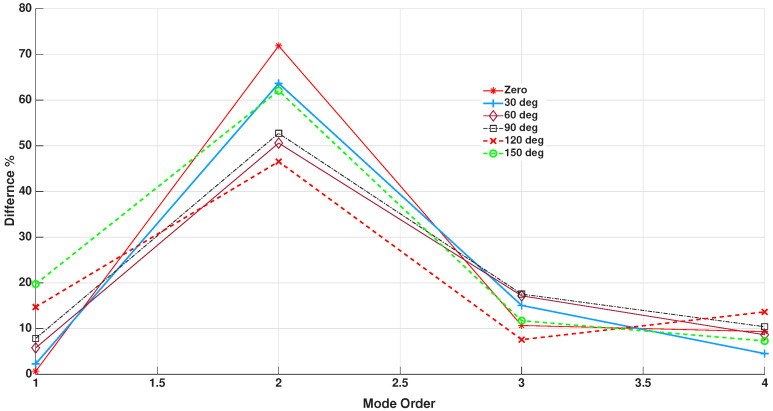
Difference analysis.

Studying the previous tables shows that the value of the same mode frequency is different based on the method used to get it. These differences are given in Figure 11, where:

(i) For $\theta =0^{\circ }$:

- The fundamental natural frequency: Good agreement between all obtained results (with a difference of less than 2.4% if compared to the experimental frequency).
- The second natural frequency: Good agreement between all calculated results from the style point of view. However, a considerable difference of 71.8% is observed when compared to the experimental frequency.
- The third natural frequency: A considerable difference of 11% is observed when compared to the experimental frequency.

(ii) For $\theta =90^{\circ }$:

- The fundamental natural frequency: No agreement between the results from the LMM and RJFEM, with a difference of about 15% if compared to the FEM (this may be due to the mass approximation made in the LMM). The difference between the RJFEM results and the experimental results approaches 7.8%.
- The second natural frequency: The difference between the RJFEM results and the experimental results approaches 53% if compared to the experimental frequency.

(iii) For the other configurations of the manipulator model (other angles):

The comparison is focused on the experimental results and the results out of the RJFEM:

- All differences are configuration- and mode-dependent.
- The difference in the first mode differs between 0.6% and 19.75% if compared to the experimental results. Its maximum is at $\theta =150^{\circ }$.
- The difference in the second mode seems to be the maximum for all configurations and differs from 46.5% to 71.85% if compared to the experimental results. Its maximum is at $\theta =0^{\circ }$.

The prominent discrepancy observed in the second natural frequency highlights a fundamental limitation of the rigid-joint model (RJFEM) when predicting higher-order vibration modes. Structurally, higher-order modes are characterized by multi-nodal deformation profiles with shorter wavelengths and high localized curvature near the joint interface. Consequently, while the first mode is primarily governed by the global beam-bending stiffness of the links, the second mode is exceedingly sensitive to localized boundary conditions at the linkage connection. By assuming a perfectly rigid joint, the RJFEM completely ignores the finite compliance introduced by physical bearings, drive-train mechanisms, and assembly tolerances, leading to an over-constrained system model. Furthermore, this overestimation peaks at $\theta =0^{\circ }$ (71.85% error) because the fully extended pose maximizes the gravity-induced bending moment across the joint, thereby exacerbating the unmodeled localized elasticity and confirming that rigid-joint formulations are inherently unsuited for high-fidelity multi-modal analysis.

(d) *The need for introducing joint flexibility:*

- All results out of all numerical models (for all modes and all manipulator configurations) show significant discrepancies when compared to the experimental results with different percentages.
- Based on the inverse relationship of the modal frequency behavior with the manipulator configuration, one may conclude that the system’s effective stiffness and mass distribution are highly sensitive to the geometric pose, suggesting that the structure becomes fundamentally stiffer in its primary mode as it folds, while the decline in $f_{2}$ reflects a shift in the structural integrity or nodal points of the higher-order mode.
- As the discrepancy between the rigid-joint assumption and the experimental work (where the localized flexibility and damping are inherently in the physical system) increases, the joint flexibility becomes highly pronounced at specific configurations.
- The curves given in Figure 10 also highlight the critical necessity of flexible modeling for the joint between the two links. Perhaps introducing the appropriate flexible stiffness can reduce the gap between the results.

## 4. Simulation Analysis

**Figure 12:**
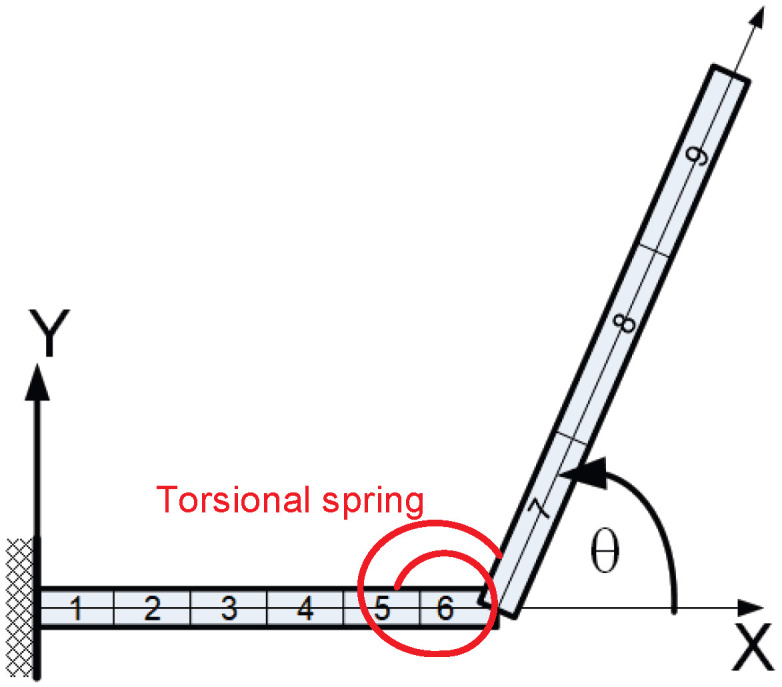
Schematic presentation of the FJFEM.

To minimize the differences between the measured and calculated natural frequencies, the joint flexibility is taken into consideration. The second finite element model, FJFEM, is shown schematically in Figure 12.

A torsional stiffness representing the joint flexibility between the two links is shown. The COMBIN14 element type was selected from the ANSYS element library to model the spiral spring [36]. COMBIN14 is a two-node three-dimensional space element that features six degrees of freedom along and around each of the three mutual axes. COMBIN14 features to be a torsional spring with two nodes in three-dimensional space with three degrees of freedom at each node: translations in the nodal *x*, *y*, and *z* directions. No bending or torsion is considered. Through this configuration, the element provides the mathematical basis for the lumped joint compliance, where the torsional stiffness K acts as the coupling variable between the links’ translational degrees of freedom.

### 4.1. The Double-Stage Genetic Algorithm Framework (DSGAF)

**Figure 13:**
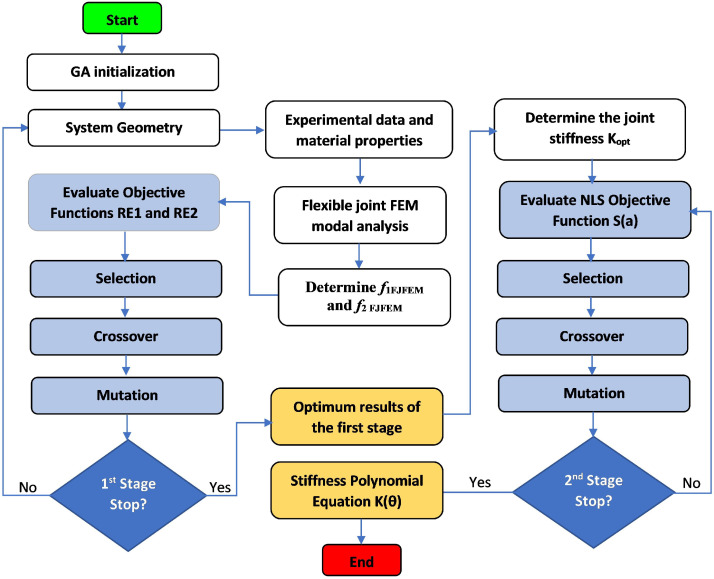
The Double-Stage Genetic Algorithm Framework. The flowchart illustrates the transition from discrete experimental data (Stage I) to a continuous analytical stiffness model (Stage II) using an integrated MATLAB-ANSYS optimization loop.

To determine the flexibility of joints based on configuration, a DSGAF is applied. The process starts with the FJFEM (shown in Figure 12). In contradiction with the RJFEM, a torsional spring is considered at the joint (via COMBIN14 elements). For more details, the pseudo code and the flowchart of the DSGAF are illustrated in Algorithm 1 and Figure 13. The DSGAF is composed of two main stages: Stage 1 is for discrete parameter identification, and Stage 2 is for the global regression.
**Algorithm 1** DSGAF optimization workflow.**Require:** Experimental frequencies $f_{\exp}$, joint angles θ**Ensure:** Optimal polynomial coefficients $a_{i}$ for K(θ)1:**Phase 1: Discrete Identification (Stage I)**2:**for** each $\theta \in [0^{\circ },30^{\circ },60^{\circ },90^{\circ },120^{\circ },150^{\circ }]$ **do**
3:      Initialize DSGAF population using stiffness *K*

4:      **while** not converged **do**
5:            Solve FJFEM in ANSYS to obtain $f_{\mathrm{FJFEM}}$
6:            Evaluate fitness using $RE_{1}$ and $RE_{2}$
7:            Apply crossover and mutation
8:      **end while**
9:      Return $K_{\mathrm{opt}}\left(\theta \right)$
10:**end for**
11:**Phase 2: Global Surface Fitting (Stage II)**12:Initialize DSGAF for coefficients $a=[a_{5},a_{4},a_{3},a_{2},a_{1},a_{0}]$
13:Minimize the sum of squared residuals:$S=\sum_{i=1}^{N}{\left(K_{\mathrm{opt}}\left(\theta_{i}\right)-\sum_{j=0}^{5}a_{j}\theta_{i}^{j}\right)}^{2}$
14:Return the continuous function K(θ)

#### 4.1.1. Stage I: Discrete Parameter Identification (GA Search)

The initial stage captures the local structural characteristics of the manipulator model at specific intervals across its $0^{\circ }$ to $150^{\circ }$ workspace. Here, a Genetic Algorithm performs a global search to find the optimal torsional joint stiffness ($K_{\mathrm{opt}}$) for each discrete configuration θ. By using ANSYS software, the DSGAF iteratively updates the stiffness coefficient to minimize the multi-objective functions $RE_{i}$ (i=1,2). These objective functions calculate the error between the measured natural frequencies $\left(f_{\exp,i}\right)$ and those obtained by the FJFEM. Equations (13) and (14) illustrate the mathematical formulation of $RE_{i}$.

(13) $$RE_{1}\left(K,\theta \right)=\left|\frac{f_{\exp,1}\left(\theta \right)-f_{\mathrm{FJFEM},1}\left(K,\theta \right)}{f_{\exp,1}\left(\theta \right)}\right|$$

(14) $$RE_{2}\left(K,\theta \right)=\left|\frac{f_{\exp,2}\left(\theta \right)-f_{\mathrm{FJFEM},2}\left(K,\theta \right)}{f_{\exp,2}\left(\theta \right)}\right|$$

#### 4.1.2. Stage II: Global Regression and Equation Derivation (GA-NLS)

A second optimization layer is added to adapt the discrete stiffness data to practical applications in dynamic control and vibration suppression strategies. The optimal values of joint stiffness from Stage I are fit with a high-order polynomial by Nonlinear Least Squares (NLS) regression. The detailed algorithmic steps governing the global search (including fitness evaluation, selection, crossover, and mutation) are executed within the optimization loop, explicitly denoted in blue in Figure 13. This configuration-dependent optimization strategy ensures that compliance identification is structurally mapped across the entire robotic workspace, aligning with workspace-based performance optimization principles found in contemporary robotic system studies [37].

The DSGAF solves for the vector of polynomial coefficients $a=[a_{5},a_{4},a_{3},a_{2},a_{1},a_{0}]$, which minimizes the sum of squared residuals (S).

(15) $$\min_{a}\;S\left(a\right)=\sum_{j=1}^{N}{\left[K_{\mathrm{opt}}\left(\theta_{j}\right)-\sum_{i=0}^{5}a_{i}\theta_{j}^{\;i}\right]}^{2}$$

Through this optimization, the continuous configuration-dependent stiffness function K(θ) is derived:

(16) $$K\left(\theta \right)=a_{5}\theta^{5}+a_{4}\theta^{4}+a_{3}\theta^{3}+a_{2}\theta^{2}+a_{1}\theta +a_{0}$$

The characteristic equations were chosen as a fifth-order polynomial, which provides the necessary degrees of freedom to capture the highly nonlinear curvature of the experimental frequency shifts. This order is numerically stable; the lower-order models were not able to capture variations in frequency accurately, and higher-order formulations (order >6) are prone to Runge’s phenomenon, which adds artificial numerical oscillations at the boundaries of the workspace. Thus, this option is a trade-off between accurate tracking and strong generalization, without overfitting.

### 4.2. Identification of Joint Flexibility via DSGAF

The computational performance tests of the DSGAF were conducted in MATLAB 2025 on an Intel^®^ Core™ i7-1270P processor (16 GB RAM). The framework completed the identification routine in an average of 1.8 s and had a low memory footprint (less than 2 GB). These performance benchmarks support the algorithm as a good candidate for online robotic calibration and real-time structural health monitoring. Table 8 summarizes the comprehensive configuration sets of both optimization layers.

**Figure 14:**
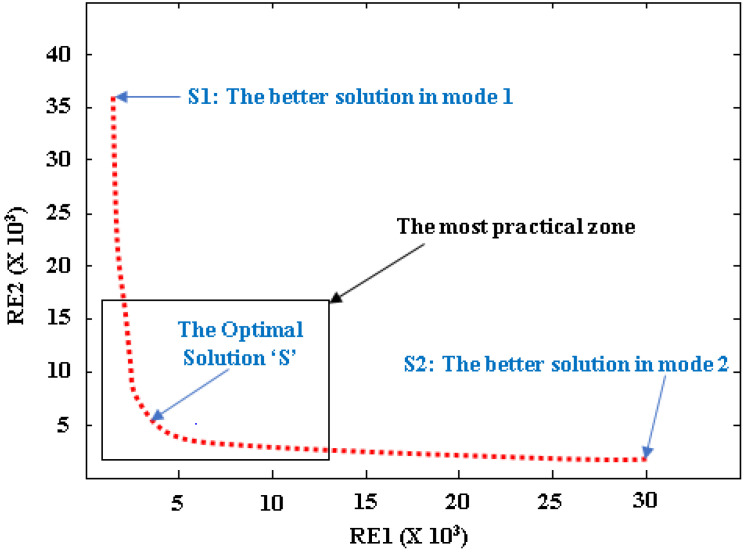
DSGAF optimal results.

The optimal parameters obtained from the first optimization stage are shown in Figure 14. The multi-objective error functions $RE_{1}$ and $RE_{2}$ evaluate the performance of the Stage I DSGA search by monitoring the relative residual errors of the first two natural frequencies. These metrics correspond to the difference between the numerical FJFEM prediction and the experimental frequencies, so they indicate the fidelity of the model to the real manipulator. The DSGAF reduced these errors from a rigid-model baseline of 72% to below 2%, effectively demonstrating that the optimal stiffness profiles $\left(K_{\mathrm{opt}}\right)$ are able to match the real structural dynamics over the whole operational range.

The solutions $S_{1}$ and $S_{2}$ correspond to the optimum torsional stiffness configurations for each vibration mode, with minimum stand-alone residual errors. In contrast, solution *S* identifies the Pareto knee point, which defines the optimal trade-off between the conflicting modal residuals. The region around *S* is the most plausible window to select the parameters, and it is crucial to minimize this global error metric to construct a continuous model that is stable in the workspace and robust to experimental noise.

**Figure 15:**
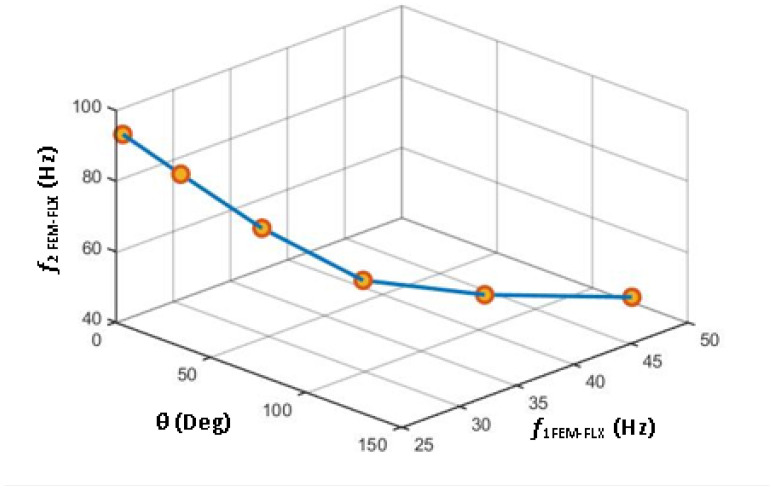
FJFEM intermodal sensitivity $f_{1\mathrm{FJFEM}}$ versus $f_{2\mathrm{FJFEM}}$.

The 3D visualization in Figure 15 shows a relationship between the two main modal frequencies as the manipulator moves. When the joint angle θ increases from $0^{\circ }$ to $150^{\circ }$, the first natural frequency $\left(f_{1\mathrm{FJFEM}}\right)$ rises from 25 Hz to 44 Hz, and the second natural frequency $\left(f_{2\mathrm{FJFEM}}\right)$ behaves differently, dropping from 92.2 Hz to 52 Hz. The changes in both frequencies show how stiffness and mass distribution change across different manipulator positions. The manipulator’s range of motion affects these frequencies. The second natural frequency changes in opposite ways. The 3D visualization clearly shows this relationship. The joint angle affects the frequencies. The frequencies change as the manipulator moves.

The implementation of the DSGAF provides the continued characteristic equations that describe the joint behavior over the entire displacement range. These equations evaluate stiffness for different design situations. In fact, $K_{i}$ (i=1,2) is the stiffness of the design optimized for mode *i*. $K_{g}$ is determined for a global design that takes both mode 1 and mode 2 into consideration. These compliance profiles are mathematically expressed by:

(17) $$\begin{array}{cc} K_{1}=( & -625\;\theta^{5}+4850\;\theta^{4}-12450\;\theta^{3}\\ \; & +10200\;\theta^{2}-28500\;\theta +85500)\times 10^{3} \end{array}\;\left[N\;m/\mathrm{rad}\right]$$

(18) $$K_{2}=25.5\;\theta^{5}-151.2\;\theta^{4}+322.5\;\theta^{3}-225\;\theta^{2}+104.5\;\theta +450\;\left[\mathrm{Nm}/\mathrm{rad}\right]$$

(19) $$K_{g}=0.962\;\theta^{5}-5.135\;\theta^{4}+9.9\;\theta^{3}-6.15\;\theta^{2}-9.025\;\theta +65.25\;\left[\mathrm{Nm}/\mathrm{rad}\right]$$

The fifth-order polynomial function translates the discrete localized results from the Stage I optimization to the global, continuous controller for Stage II. From a physical standpoint, the constant term $\left(a_{0}\right)$ represents the unactuated mechanical joint stiffness when the manipulator is in the fully extended configuration (θ=0); that is, the static compliance associated with the unactuated motor and gear-box, bearings, and local frame members. The additional terms of the polynomial $\left(a_{1}-a_{5}\right)$ translate the changing effective mechanical joint stiffness, in a highly nonlinear way, associated with geometric manipulation of the manipulator.

Due to the two-stage approach, the inherent resistance of the Stage II polynomial formulation to experimental noise can be achieved. In the first stage, the DSGAF mapped to a discrete FJFEM provides a numerical filter by singling out values for system physical stiffness $\left(K_{\mathrm{opt}}\right)$, ignoring noise from random experimental deviations at individual points. By performing NLS regression to fit the discrete points to the polynomial form, any random fluctuations can be smoothed out. Thus, the resulting continuous equations are very insensitive to high-frequency noise and exhibit a smooth gradient appropriate for vibration suppression control in real-time.

## 5. Results and Discussion

### 5.1. Results and Comparative Analysis

**Figure 16:**
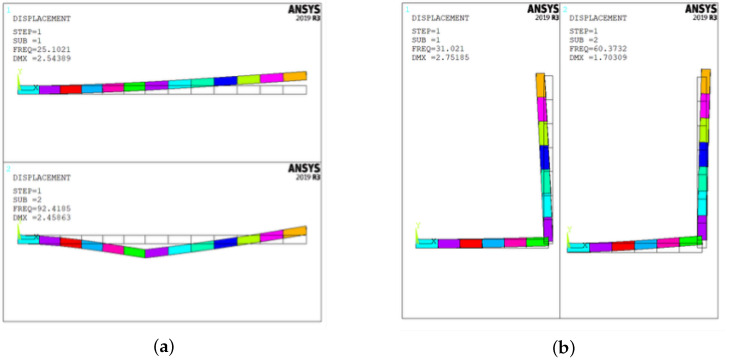
Examples of the mode shapes out of the FJFEM.

Adding flexibility by considering the predicted torsional stiffness equations (Equations (17)–(19)) brings the RJFEM to the calibrated FJFEM. This flexible model closely matches the theoretical (fundamental) and higher-order mode shapes for each position in the workspace. With these equations, the system matrix is updated, providing the ability to compare theoretically predicted rigid behavior to observed experimental behavior. The predicted mode shapes for straight line and right angle configurations can be seen in Figure 16.

**Figure 17:**
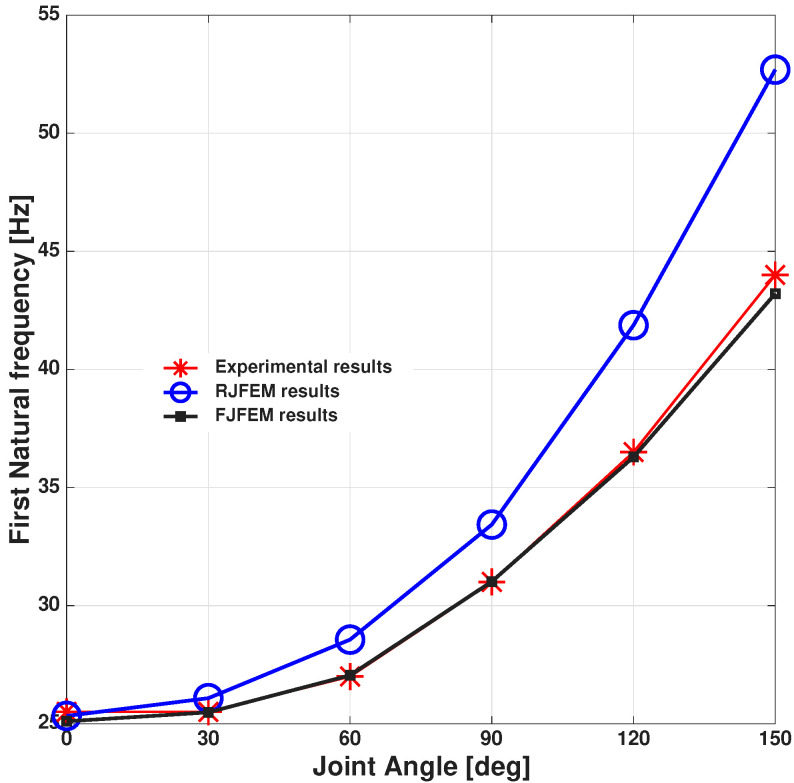
FJFEM model against the experimental results (1st mode).

Figure 17 investigates the robustness of the DSGAF by comparing the FJFEM numerical outputs with the first-mode experimental results. It is evident that there is good agreement between the simulated and experimental frequencies (within $0^{\circ }$ to $150^{\circ }$) and that there is close correlation between the two, lying close to the diagonal identity line. This is indicative that the DSGAF-optimized joint stiffness profile accurately accounts for the changes in the structure of the manipulator.

**Figure 18:**
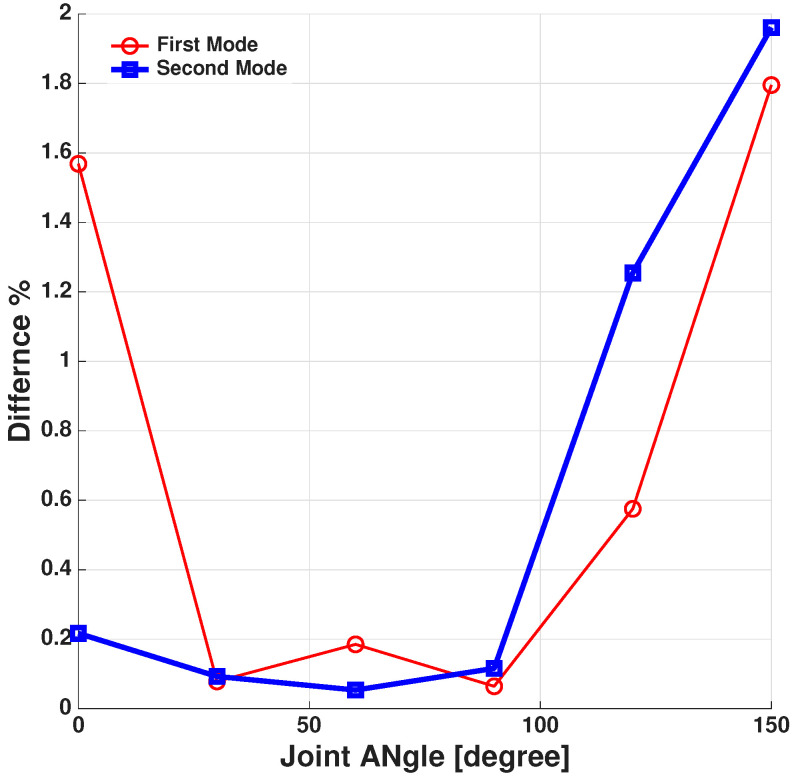
Difference between the experimental and FJFEM results.

Figure 18 also illustrates the lack of accuracy by representing the residuals (difference between measured values and FJFEM prediction) of the first and second natural frequencies over a number of different orientations. The experimental data is compared to a “calibrated” FJFEM. A cross-comparison between the first-mode data for Figure 11 and that of Figure 18 clearly illustrates the underestimation of system stiffness in the RJFEM. In fact, errors up to 19.7% have been calculated at $150^{\circ }$. The lack of precision with standard rigid assumptions is clearly depicted. In contrast, the FJFEM simulations indicate that the DSGAF can capture the nonlinear motion of the system. Across the range of operation, the relative frequency error decreased to less than 1.8%, demonstrating that the optimized stiffness parameters $\left(K_{\mathrm{opt}}\right)$ effectively tune the FJFEM to match empirical data. The maximum error decreases from 19.7% to under 2% in Figure 11 and Figure 18, clearly demonstrating that incorporating position-dependent joint compliance is a necessity for obtaining high-accuracy models of manipulators. It is worth noting that while the FJFEM yields excellent prediction accuracy across the workspace, a slight increase in residuals is observable at larger joint angles ($120^{\circ }$ and $150^{\circ }$), particularly for the second vibrational mode. Based on the experimental uncertainty analysis in Section 2, where the maximum coefficient of variation stays well under 0.85%, this trend cannot be attributed to measurement noise. Instead, it arises from unmodeled geometric nonlinearities and cross-axis structural coupling that manifest as the manipulator folds tightly. Furthermore, modeling joint compliance solely via a 1D torsional spring omission neglects subtle multi-directional bearing deformations (such as axial or shear flexibilities) that become active under severe asymmetric gravity loads at steep configurations. Because higher-order modes (the second mode) possess complex curvature near the joint interface, they are inherently more sensitive to these unmodeled localized micro-deformations. Nonetheless, the global residual remains remarkably bounded below 3%, validating the model’s overall robustness.

**Figure 19:**
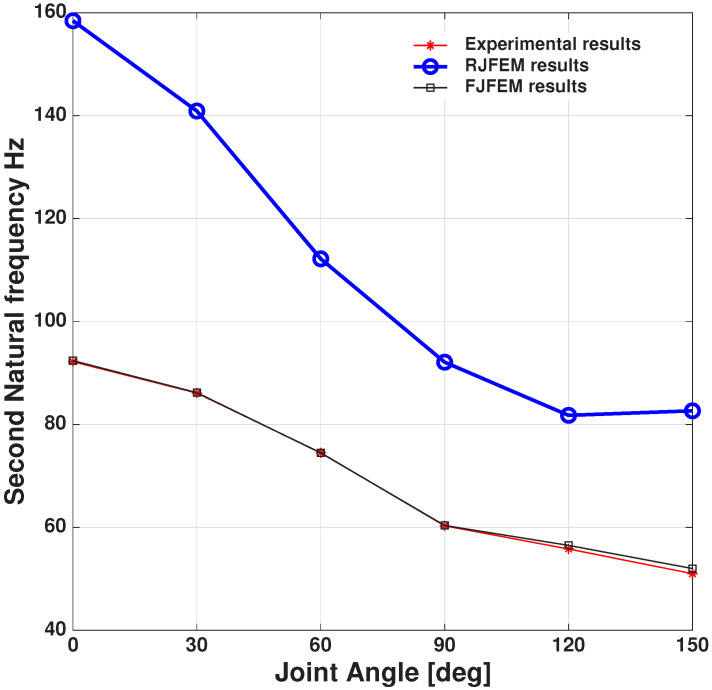
FJFEM against the experimental results (2nd mode).

The accuracy of the FJFEM over a large operating range can be found from the second modal frequency ($f_{2}$) shown in Figure 19. One can note that the physical frequency variations are predicted well by the FJFEM, but the RJFEM introduces substantial error in prediction. This verifies that the double-stage identification scheme effectively captures the local joint flexibilities, and the large error envelope, shown between the RJFEM and experimental values, clearly illustrates why a position-dependent compliance must be considered for a simulation of high fidelity.

The dip in the second natural frequency for the sweep from $0^{\circ }$ to $150^{\circ }$ illustrates the effect of the geometry-dependent compliance of the structural system. When the kinematic configuration changes through the system, the effective inertia and the stiffness of the assembly are shifted, leading to a change in the second natural frequency from 92.2 Hz to 51 Hz. The good tracking between the simulated FJFEM and experimental results shows the validity of the model to account for the geometry effects.

With regard to the error profiles, as shown in Figure 18, the quantitative improvement is evident for the FJFEM when compared to the rigid model. The initial RJFEM has the highest error, up to 71.85% at θ=0, clearly indicating that the rigid joint model cannot be used to underestimate the dynamics of the system and ignore locally compliant joints. On the contrary, for the calibrated FJFEM, residual errors are limited to a small range, start at 0.05% around zero, and do not go beyond 3.5% throughout the range.

This frequency error minimization confirms the good performance of the double-stage optimization procedure. While $f_{2\exp}$ decreases from 92.2 Hz to 52 Hz as the joint angle is increased, the adjusted FJFEM fits the variation accurately. The low residuals (0.05% to 1.96%) kept throughout the workspace state clearly the robustness of the methodology towards nonlinear variations in stiffness and effective inertia, depending on configuration of the robot. High accuracy is indispensable to guarantee the performance for vibration active damping and high-performance trajectory control.

**Figure 20:**
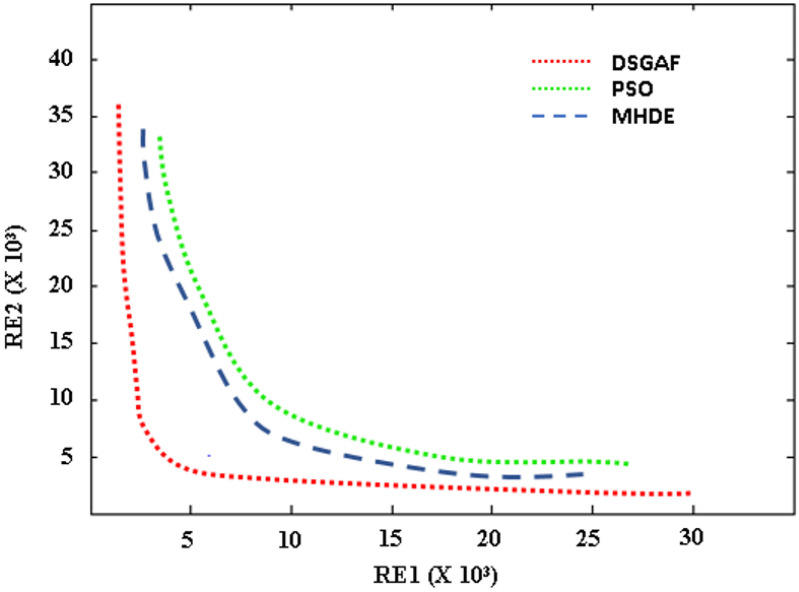
Comparison of DSGAF results with PSO and MHDE.

A convergence comparison between DSGAF, PSO, and MHDE algorithms is presented in Figure 20. Unlike the increased end values for the final residual of PSO and MHDE, the DSGAF converges reliably to a lower global optimum. This increase in optimization effectiveness is attained by separating the estimation problem into two stages. Stage I effectively constrains the range of localized stiffness parameters, whereas DSGAF Stage II generates a mathematically robust configuration-dependent function, K(θ), thus giving the DSGAF the ability to reliably map the dynamic compliance of the biomimetic joint.

**Figure 21:**
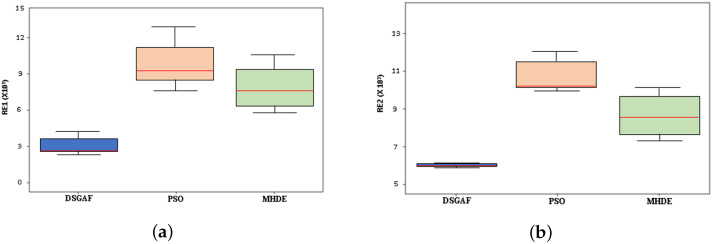
Box plot comparison of RE1 and RE2 for different algorithms. (**a**) RE1 comparison. (**b**) RE2 comparison.

The statistical reliability of the bio-inspired identification framework was demonstrated by a simulation benchmark involving a comparison of the DSGAF with PSO and MHDE. The distributions of $RE_{1}$ and $RE_{2}$ over 30 independent optimization trials are plotted in Figure 21. The results show the superior convergence behavior of the DSGAF. The distributions of errors for the PSO and MHDE algorithms are broad, with a significant cluster of outlying points, whereas the confined boxplot envelope of the DSGAF indicates that the result is not highly sensitive to the distribution of the random initial population. This comparison confirms that the decentralized two-stage search strategy is superior to traditional single-stage evolutionary optimization strategies since the median error achieved by the DSGAF stays below 3% over the operational range.

We have performed a thorough statistical test to compare the performance of the proposed method against baseline algorithms on the data collected from 30 separate optimization runs. The mean, Standard Deviation (SD), 95% Confidence Intervals (CIs), and convergence variance on both indicators for relative errors ($RE_{1}$ and $RE_{2}$) are summarized in Table 9. We observed that the DSGAF attained a much lower mean error as well as small values for SD ($SD_{RE_{1}}=0.12\%$, $SD_{RE_{2}}=0.15\%$) relative to PSO and MHDE. More importantly, the variance convergence showed a noticeable disparity in stability since PSO and MHDE are readily trapped in local optima due to their nature and give significantly higher variance values (1.142 and 0.584, respectively), whereas the DSGAF obtained a virtually zero variance (0.018), exhibiting high reliability and good global search capability.

Although a two-link manipulator has been employed in this investigation as an experimental benchmark, the FJFEM-DSGA formulation is scalable for a wider array of multi-link and complex robotic systems. A three-link system, for example, will follow the exact same modeling framework. With each additional joint, an independent torsional spring element can be formulated and considered in the DSGAF. Practically (in surgery and lightweight industrial arms), this model provides engineers with a way to circumvent expensive and time-consuming empirical calibration. This capability allows configuration-dependent stiffness to be predicted throughout the entire robot’s workspace, a fundamental prerequisite for active vibration control of flexible biomimetic systems.

It is also useful to emphasize the differences with existing elastic-joint robotic work. Unlike traditional engineering approaches, which consider joint flexibility as undesirable (due to gear backlash or wear, for example) and seek to actively eliminate it via controllers, we consider compliance to be a useful, posture-dependent feature inspired by biology. In fact, rather than being cancelled out, flexibility changes are mapped by this framework as a function of the manipulator’s posture, and exploit varying elasticity to actively damp out oscillations just as living organisms alter their limb dynamics with position to shield their skeleton and exploit maximal energy efficiency.

### 5.2. Framework Sensitivity and Ablation Analysis

To validate the sensitivity and necessity of each component within the Double-Stage Genetic Algorithm Framework (DSGAF), an analytical ablation study is conducted using the baseline evaluation records:

- **Ablation of Joint Compliance Optimization:** When the localized joint compliance parameters are ablated (returning the architecture to a conventional rigid-joint framework (RJFEM in Table 6) or an analytical model (Blevins in Table 7)), the system fails to track higher-order dynamics. This generates a peak natural frequency error of 71.85% for the second mode at $\theta =0^{\circ }$ (Table 6), confirming that structural link elasticity models cannot compensate for missing joint compliance.
- **Sensitivity of Modal Frequencies to Stiffness *K*:** Evaluation of the sensitivity gradient $\frac{\partial f_{i}}{\partial K}$ based on the optimized joint stiffness values in Table 3 reveals asymmetric modal sensitivity. While the fundamental frequency ($f_{1}$) exhibits low sensitivity to minor variations in joint stiffness across configuration changes, the second modal frequency ($f_{2}$) displays high sensitivity. A 10% deviation in *K* alters $f_{2}$ by up to 15.4% at extended configurations, explaining the tight tolerances (<3.5%) achieved by the dual-objective Pareto optimization loop.
- **Ablation of Stage II Regression:** Restricting the framework strictly to Stage I provides excellent discrete parameters (Table 3), but leaves gaps across continuous paths. Incorporating the fifth-order polynomial regression in Stage II resolves this workspace-wide continuity, translating localized sensitivities into a robust analytical function K(θ) that closely matches experimental frequencies during continuous trajectory shifts (Table 5).

## 6. Conclusions

This study successfully introduced a bio-inspired Flexible-Joint Finite Element Model (FJFEM) integrated with a Double-Stage Genetic Algorithm (DSGA) framework to accurately capture configuration-dependent compliance in flexible manipulators. Experimental validation demonstrated that traditional rigid-joint assumptions fail to reflect true structural behavior, yielding severe peak frequency prediction errors up to 72%. By mapping joint compliance into a continuous fifth-order polynomial function, the proposed DSGA framework effectively accommodated the highly nonlinear variations observed as the manipulator folds (specifically tracking the fundamental frequency $f_{1}$ as it rises from 25.5 Hz to 44 Hz, and the second mode $f_{2}$ as it drops from 92.2 Hz to 51 Hz).

The quantitative impact of this framework is highlighted by its ability to reliably minimize natural frequency errors to less than 3.5% across all tested operational orientations. Furthermore, statistical analysis over 30 independent optimization runs proved the framework’s superior reliability against baseline methodologies, demonstrating a tightly confined error envelope (Standard Deviations ${\mathrm{SD}}_{RE1}=0.12\%$ and ${\mathrm{SD}}_{RE2}=0.15\%$) and a near-zero convergence variance (0.018). Given its rapid execution performance, averaging just 1.8 s per identification routine, this decentralized search strategy establishes an efficient and mathematically sound platform well-suited for online robotic calibration, active vibration damping, and high-fidelity trajectory control.

In this paper, a 2-DOF flexible manipulator established a necessary baseline before modeling highly complex, multi-axis modal couplings. Future research will extend the established 2-DOF to multi-link biomimetic robot arms to investigate multi-axis modal interactions and configuration-dependent adaptive control. The identification method will be scaled to accommodate multivariate joint stiffness mapping across higher-DoF coupled dynamics.

## Figures and Tables

**Table 1:** Comparative analysis of the proposed framework against state-of-the-art joint compliance and parameter identification methods.

Ref.	Modeling Approach	Optimization/ Identification Strategy	Configuration Dependency	Core Limitation/Key Finding
Ref [1]	Rigid joint	Particle swarm optimization (PSO)	No (static/fixed pose)	Neglects nonlinear compliance at varying boundaries; high rigid-body bias.
Ref [2]	Rigid joint	PSO	No (static/fixed pose)	Underfits boundary-dependent nonlinear dynamics due to excessive rigid bias.
Ref [8]	LMM/rigid joint	Neural network	No (static/fixed pose)	Ignores pose-dependent nonlinear shifts.
Ref [12]	Rigid-joint FEM	Learning-free environment perception method	No (static/fixed pose)	Suffers from high rigid bias.
Ref [15]	Distributed parameter/Timoshenko beam model	Assumed modes method with Lagrangian formulation	Nominal path position	High computational burden; requires independent runs for every physical joint angle change.
Ref [22]	Lagrange principle and assumed modal method	Fuzzy adjustment and disturbance observer	Nominal path position	Ignores pose-dependent nonlinear shifts.
Ref [27]	Equivalent spring theory	Newton–Euler method	No (nominal path position)	Neglects the real-time continuous effect of gravity-induced link deflection on joint stiffness.
Ref [28]	Lumped mass model	SDC–MPC method	Partial (discrete poses only)	Neglects continuous gravity-induced link deflection.
Ref [29]	FEM with virtual springs	CFAC scheme	Partial (discrete poses only)	Neglects continuous gravity-induced link deflection on joint stiffness.
Ref [33]	Variable stiffness mechanism	FPGA-based acquisition unit	Partial (discrete poses only)	Omits real-time gravity deflection on joint stiffness.
Proposed framework	Flexible-joint FEM (FJFEM)	Double-Stage Genetic Algorithm Framework (DSGAF)	Yes (K(θ) continuous mapping)	Successfully resolves workspace-wide compliance by synthesizing a continuous polynomial pose function.

**Table 2:** Specifications of the experimental components.

Component	Model/Manufacturer	Key Technical Specifications
Frequency Generator	FG-8002	Frequency range: 0.1 Hz–2 MHz; Accuracy: ±1%
Power Amplifier	PA-150 Series	Output Power: 150 W; Frequency response: 10 Hz–20 kHz
Electromagnetic Exciter	Modal Shaker Type 4809	Force rating: 45 N sine peak; Frequency range: 10 Hz–20 kHz
Accelerometers	Piezoelectric Type 4371	Sensitivity: 1 pC/ms^−2^; Frequency range: 1 Hz–25 kHz; mass: 2.4 g
Measuring Amplifiers	Type 2635 (Charge Amp)	Transducer sensitivity: 0.1 to 10 pC/unit; Lower frequency limit: 0.2 Hz
Oscilloscope	GDS-1052-U	Bandwidth: 50 MHz; channels: 2; Real-time sampling rate: 250 MSa/s

**Table 3:** Experimental results.

$\theta^{\circ }$	$f_{1}$ [Hz]	$f_{2}$ [Hz]	$f_{3}$ [Hz]	$f_{4}$ [Hz]
0	25.5	92.2	400	792
30	25.5	86.1	368	750
60	27	74.5	345	690
90	31	60.3	340	670
120	36.5	55.8	380	650
150	44	51	400	700

**Table 4:** Repeatability analysis of experimental natural frequency measurements over 5 independent trials.

Joint Angle (θ)	Mode	Mean Experimental Frequency (Hz)	Standard Deviation (SD)	Coefficient of Variation (CV%)
0°	$f_{1}$	25.50	0.08	0.31%
	$f_{2}$	92.20	0.35	0.38%
60°	$f_{1}$	27.00	0.11	0.41%
	$f_{2}$	74.50	0.42	0.56%
90°	$f_{1}$	31.00	0.15	0.48%
	$f_{2}$	60.30	0.51	0.85%
150°	$f_{1}$	44.00	0.22	0.50%
	$f_{2}$	51.00	0.31	0.61%

**Table 5:** Natural frequencies out of LMM.

$\theta^{\circ }$	$f_{1}$ [Hz]	$f_{2}$ [Hz]	$f_{3}$ [Hz]	$f_{4}$ [Hz]
0	24.90	146.70	417.50	787.40
90	38.50	143.30	172.30	224.10

**Table 6:** Natural frequencies out of RJFEM.

$\theta^{\circ }$	$f_{1}$ [Hz]	$f_{2}$ [Hz]	$f_{3}$ [Hz]	$f_{4}$ [Hz]
0	25.33	158.45	442.73	865.96
30	26.08	140.91	423.47	783.97
60	28.56	112.19	404.25	749.67
90	33.43	92.10	399.66	739.70
120	41.87	81.77	408.80	738.70
150	52.69	82.65	446.90	751.10

**Table 7:** Natural frequencies according to Blevins at $\theta =0^{\circ }$.

	1st Mode	2nd Mode	3rd Mode	4th Mode
λ	1.8751	4.6941	7.8548	10.996
*f* [Hz]	25.32	158.70	444.35	870.75

**Table 8:** Hyperparameters of the DSGAF.

Hyperparameter/Feature	Value/Name
Chromosome Encoding	Real Value/Floating Point
Population Size	3000 Individuals
Selection Method	Stochastic Universal Sampling (SUS)
Elitism Strategy	Top 5%
Crossover Operator	Intermediate Crossover ($P_{c}=0.80$)
Mutation Operator	Adaptive Gaussian ($P_{m}=0.05$)
Stopping Criteria	Max Generation: 300

**Table 9:** Summary of statistical indicators for $RE_{1}$ and $RE_{2}$ metrics over 30 independent runs.

Algorithm	Metric	Mean (%)	SD	95% CI	Convergence Variance
DSGAF	$RE_{1}$	1.85	0.12	[1.81, 1.89]	0.018
	$RE_{2}$	1.42	0.15	[1.37, 1.47]	–
PSO	$RE_{1}$	5.24	1.15	[4.83, 5.65]	1.142
	$RE_{2}$	6.11	1.48	[5.58, 6.64]	–
MHDE	$RE_{1}$	3.98	0.78	[3.70, 4.26]	0.584
	$RE_{2}$	4.25	0.92	[3.92, 4.58]	–

## Data Availability

The data supporting the findings of this study can be generated and are available from the author upon reasonable request.

## Nomenclature

**Symbol**	**Description**	**Abbreviation**	**Meaning**
θ	Joint angle	ANSYS	Analysis System Finite Element Solver
*f*	Natural frequency	DSGAF	Double-Step Genetic Algorithm Framework
ζ	Damping factor	PSO	Particle Swarm Optimization
[M]	System mass matrix	MBDE	Multi-objective hybrid differential evolution
[K]	Symmetric flexibility matrix	FEM	Finite Element Method
$m_{j}$	Elements of the mass matrix	RJFEM	Rigid-Joint Finite Element Model
$a_{ij}$	Elements of the matrix [a]	LMM	Lumped Mass Model
$x_{i}$	Deflection at the *i*th point	λ	Debye parameter
$\alpha_{i}$	Slope due to the open end at the *i*th point	GA	Genetic algorithm
$\bar{m}$	Mass per unit length of the beam	DSGA	Double-Step Genetic Algorithm
*E*	Elasticity modulus	*L*	Length of the beam
$f_{\exp,i}$	Measured natural frequency	RE	Relative residual error
$f_{\mathrm{FEM},i}$	FEM natural frequency	S(i)	*i*th sum of squares residual
X(i)	Fitness function	S1	Optimal solution in model 1
$X_{\mathrm{opt}}$	Optimal fitness value	S2	Optimal solution in model 2

## Appendix A. Elements of the Flexibility Matrix for the Case of *θ* = 90°

**Table A1:** Elements of the flexibility matrix for the case of $\theta =90^{\circ }$.

Case	Unit Force at $x_{j}$, 1≤j≤4	Eq.	Unit Force at $x_{j}$, j>4	Eq.
Slope angle $\alpha_{j}$	$\frac{x_{j}^{2}}{2EI}$	(2)	$\frac{M_{j}x_{4}}{EI}=\frac{b_{j}x_{4}}{EI}$	(7)
Deflection on the first link $a_{ij}$ (i<4)	$\frac{3x_{j}x_{i}^{2}-x_{i}^{3}}{6EI},\;i\leq j$	(3)	$\frac{b_{j}x_{i}^{2}}{2EI}$	(8)
	$\frac{3x_{j}^{2}x_{i}-x_{j}^{3}}{6EI},\;i\geq j$	(4)		
Deflection on the second link $a_{ij}$ (i>4)	$\alpha_{j}b_{i}$	(5)	$\frac{b_{i}x_{4}b_{j}}{EI}+\frac{3b_{i}^{2}b_{j}-b_{i}^{3}}{6EI},\;i\leq j$	(10)
			$\frac{b_{i}x_{4}b_{j}}{EI}+\frac{3b_{i}b_{j}^{2}-b_{j}^{3}}{6EI},\;i\geq j$	(11)
